# The experiences of female community health workers during COVID-19 and the influence of gender norms and values in Nepal: A qualitative study

**DOI:** 10.1371/journal.pgph.0006393

**Published:** 2026-05-06

**Authors:** Abriti Arjyal, Sushil Baral, Sulata Karki, Ayuska Parajuli, Obindra Bahadur Chand, Shophika Regmi, Wesam Mansour, Sally Theobald, Sophie Witter, Joanna Raven

**Affiliations:** 1 HERD International, Lalitpur, Nepal; 2 Liverpool School of Tropical Medicine, Liverpool, United Kingdom; 3 School of Health and Social Care, University of Essex, Colchester, United Kingdom; 4 Institute for Global Health, Queen Margaret University, Edinburgh, United Kingdom; University of Washington Department of Global Health, UNITED STATES OF AMERICA

## Abstract

Female Community Health Volunteers (FCHVs) are close-to-community providers who voluntarily serve their communities in Nepal. Gaps exist in evidence regarding the challenges posed by shocks such as the pandemic, as well as the gendered experiences of FCHVs in the general context. This study aimed to explore the experiences and challenges faced by FCHVs during COVID-19 and the influence of gender norms and values. We conducted a qualitative study in two municipalities, Gulariya in Lumbini province and Chandragiri in Bagmati province, Nepal. A total of 22 interviews were conducted between December 2020 and January 2021 using topic guides. These included key informant interviews with purposively sampled health coordinators, health facility managers, and local mayors, as well as in-depth interviews with FCHVs. A thematic framework approach, incorporating a gender framework, was applied for data analysis. NVivo 11 Pro software was used to support the analysis. FCHVs faced significant challenges due to gender norms, family expectations, and insufficient support from the health system during COVID-19. Balancing dual responsibilities at home and work created further difficulties in their daily lives. Although FCHVs were from and embedded within their communities and gained trust over time, they experienced some unsupportive behaviours, particularly from men who were not receptive to their roles. Inadequate support, such as a lack of supervision and training on COVID-19, further undermined the confidence of FCHVs and affected their ability to provide services. Shortages of personal protective equipment, lack of financial incentives, and male-dominated leadership hindered gender-sensitive planning, exacerbating the challenges FCHVs faced during the crisis. FCHVs are vital to Nepal’s health system, especially during crises like COVID-19. Addressing gender norms, providing tailored training, promoting inclusivity, and involving male volunteers are key policy priorities. Future efforts should focus on strengthening FCHVs’ resilience and addressing societal gender challenges.

## Background

In many Global South nations, particularly in fragile and shock-prone environments, the lack of appropriately trained healthcare personnel is a major obstacle to providing essential health services [1]. To address this gap and provide services to the most disadvantaged groups, there is an increasing dependence on a variety of Close-to-Community (CTC) providers, including community health workers (CHWs), auxiliary nurse midwives, health auxiliaries, family welfare educators, volunteers, village health workers, community health aides, barefoot doctors, traditional healers, and certified birth attendants [1,2]. CTC providers are community health workers or volunteers who function as the main point of contact with the community, carrying out health promotion, preventative, and/or curative treatments [3]. These CTCs are essential for progressing efforts towards universal health coverage [4].

There is diversity of CTC provider roles across countries and the challenges they faced which are also shaped by gender norms. For example, in Bangladesh, informal CTC provider largely women are widely accepted in urban slums, for their perceived advantages in availability, accessibility, and affordability making them first point of contact for many seeking sexual and reproductive health services despite lacking formal training [5]. In African countries such as Malawi, both male and female CTC providers are present, with men often involved in community activities that require physical strength or leadership. They are responsible for tasks like community mobilization, health education, disease surveillance, and vector control activities including environmental management and fogging [6,7]. In certain situations male CTC providers can more successfully engage with male groups or deal with certain health issues that female volunteers would find challenging, particularly where women’s mobility may be limited due to cultural restrictions [8].

In low- and middle-income countries (LMICs), studies have identified a preference for women to take on the CTC provider roles, particularly among women, as it creates a comfortable environment for them to discuss their health-related issues [9–11]. However, female CTC providers faced a higher vulnerability to both verbal and physical assault. Despite facing challenges in attaining a work-life balance, adequate resources, training, compensation and supportive supervision, they remain committed to enhancing the health of their communities [6]. In specific locations in LMICs, pairing male and female CTC providers was recommended to enhance the safety of women [12].

**Box 1. Female Community Health Volunteers in Nepal**

In Nepal, FCHVs serve as CTC providers, community health cadres working voluntarily in all 77 districts. The ‘Female Community Health Volunteer Programme’ initiated by the Government of Nepal (GoN), began in 1988/1989 in 27 districts, expanding nationwide. Given the GoN’s focus on prevention and promotion of child, maternity, newborn, and reproductive health services through this initiative, only female volunteers were accepted. Currently, there are 51,416 active FCHVs in Nepal [13]. Members of the Mothers’ Group for Health in collaboration with local healthcare facility staff identify FCHVs based on national guidelines. With changing guidelines, current selection criteria include being a long-term resident of the community willing to serve for at least five years; being aged between 25 and 45; and minimum education requirement of grade 10, except in mountainous and rural areas, where FCHVs should have elementary education [14]. FCHVs work closely with their community and local health facilities, by coordinating with supervisors who can be who are either the person in charge of the health post, nurses, or auxiliary health workers [15].

In Nepal, the experiences of FCHVs, who are CTC providers are indicative of gendered social norms and power dynamics. Box 1 provides details of the FCHV programme in Nepal. FCHVs, by policy are all women, and they often deal with issues such as intimidating behaviour from men, which can lead to stress and fear, especially when walking long distances alone [16]. FCHVs have effectively built rapport and trust with the people in their catchment area due to their commitment to serving their communities [17,18]. They are also deeply rooted in the sociocultural norms and values of their local communities, making it easier for them to volunteer in community health and understand their contexts [19].

There are gaps in evidence in how best to adapt policy and practice (e.g., support structures) to address challenges posed by shocks such as the pandemic and the gendered experiences of CTC providers during such crises [20]. Nepal ranks fourth, eleventh, and thirtieth in terms of vulnerability to earthquake, flood, and climate change risks respectively [21]. Additionally, its open border with India makes it highly susceptible to the cross-border spread of diseases, as evidenced by the simultaneous peak incidence of COVID-19 cases in China and neighbouring India [22]. The recent decentralization, characterized by unclear roles and limited capacity among new local governments, intensifies fragility in the healthcare system, creating challenges in the execution and implementation of health services within the restructured framework [23,24].

While previous studies have emphasized the important roles FCHVs play in combating gender-based violence and supporting maternal and child health, there is a scarcity of literature concerning the gendered experiences of FCHVs in Nepal. Furthermore, a study identified the vital role that CTC providers play in pandemics, stressing the necessity for additional research on gender and equity, as well as emphasizing the importance of role clarity, training, supportive supervision, and their health and well-being during pandemics and shocks [25]. To strengthen the response to future shocks such as COVID-19 and crises, it is imperative to analyse CTC provider’s experience from a gender perspective and develop equitable and inclusive strategies [26]. The ReBUILD for Resilience research programme concentrates on enhancing health systems resilience in fragile settings, including in Lebanon, Myanmar, Nepal, and Sierra Leone. As an integral component of this programme, a research study was conducted to explore the gendered experiences of CTC providers in these countries during the COVID-19 pandemic [27]. In this paper, we explore FCHVs’ experiences and challenges faced in Nepal while delivering health care services during COVID-19 and the influence of gender norms and values. This study highlights how FCHVs managed overlapping responsibilities and strategies, and contributes to the literature by providing in-depth insights into how gender norms, local social expectations, and pandemic-related shocks influenced FCHVs’ work in Nepal.

## Methods

### Study design

We used qualitative research methods (key informant interviews with decision makers and managers, and in-depth interviews with FCHVs at the municipality level) to explore FCHVs’ experiences during COVID-19 and the influence of gender norms and values. This generated in-depth and contextual information about FCHVs’ and other stakeholders’ experiences and perceptions and explored the reasons behind their answers through probing questions [28,29].

### Study setting

In Nepal the first case of COVID-19 was reported on January 13, 2020 [30]. The Government of Nepal (GoN) imposed a state wide lockdown from March 24 to July 21, 2020, restricting non-essential services and travel, both domestically and internationally [22]. Only two COVID-19 infections were documented during the lockdown, and no deaths were linked to the virus [5]. In June 2020, there was a spike in COVID-19 cases despite the lockdown, which resulted in community-level transmission across the nation. When this study was conducted, all districts were impacted; and 1,003,450 cases and 12,031 deaths overall were reported [31]. The Kathmandu valley was a hotspot during the pandemic, and Chandragiri is one of the municipalities within the valley. At the beginning of December 2020, Kathmandu reported 12,000 confirmed cases with a considerable number of active cases. During this period, cases also increased in Lumbini Province, which includes Gulariya municipality in Bardiya district. By mid-December 2020, the province had recorded a significant rise in cases (test positivity rate to 24.3%), while the national positivity rate averaged 15.6% [32]. We conducted the study in two municipalities: Gulariya, Lumbini province and Chandragiri of Bagmati province. These sites reflect diverse socio-demographic, geographic, and health system context within Nepal. Gulariya municipality consists of 12 wards and lies in the western plains of Nepal and shares a border with India. The municipality has a diverse caste and ethnic composition with people from ethnic groups such as Tharu, Madhesi, Muslim, and a hill group known as ‘*pahade’* [33]. According to the 2021 National Census by the Central Bureau of Statistics, Gulariya municipality had approximately 16 thousand households with a total population of 74,505 of whom 49.3% (36,731) were male and 50.7% (38,774) were female [34]. Gulariya municipality directly borders India’s Uttar Pradesh state. During the pandemic period, India had also implemented lockdown which forced Nepali migrant workers returned to home country [35].

Chandragiri municipality is situated in the Southwestern part of Kathmandu valley and consists of 15 wards. It has a diverse caste and ethnic composition with Newar being the majority followed by Chhetri, Brahmin, Tamang and Magar [36]. It had approximately 36 thousand households with a total population of 136,860 of whom 49.9% (68,293) were male and 50.1% (68,567) were female [34].

The health system context of two municipalities also differs. Chandragiri situated within the capital city (Kathmandu), benefits from proximity to tertiary and specialised hospitals, whereas Gulariya relies more on decentralized province level health infrastructures. We selected these two municipalities to understand FCHVs’ experiences of working during the pandemic in different geographical and socio-cultural contexts. We had previously worked in these districts and had well-established networks and relationships that enabled access and data collection during the COVID-19 pandemic. Within these two municipalities we selected six wards based on discussions with local stakeholders including health coordinators and the COVID-19 focal person of the municipality, ensuring geographical representation, socio-cultural diversity, and diverse ethnic and linguistic participation.

### Study participants

We conducted interviews with 13 FCHVs and 9 key informants across two municipalities. All the FCHVs were married with ages ranging from 29 to 58 years and education background from illiterate to intermediate levels. The duration of their volunteer work varied from 3 to 30 years, indicating a mix of experienced and newer volunteers. The FCHVs came from diverse ethnic groups. In Gulariya (Bardiya district), participants belonged to Brahmin, Madhesi, Chaudhari, Janajati, Muslim, and other groups, while in Chandragiri (Kathmandu district), the FCHVs included Dalit, Brahmin, Newar, Sanyasi (Giri), and other Janajati groups. Key informants included health post in-charges, health coordinators, and local government officials, with diverse ethnic backgrounds and varying years of work experience. The details about participants are attached in S1 Table.

## Interviews

### Sampling

We identified FCHVs for in-depth interviews with the support of the health facility in charges. We obtained lists of FCHVs for the selected wards and purposively selected FCHVs from these lists considering diversity in socio-cultural background, caste-ethnic composition, duration of work experiences, education, and active engagement while serving the community during COVID-19.

We selected key informants based on their knowledge and experiences about FCHVs’ work and their engagement with FCHVs and COVID-19 response and management. The key informants included health coordinators, health facility managers and mayors – elected political lead of the municipality (Table 1).

**Table 1:** Interview details.

	Gulariya municipality	Chandragiri municipality	Total
In-depth interview (IDI)	FCHVs (n = 7)	FCHVs (n = 6)	13 (F)
Key informant interview (KII)	Sub health coordinator (1)	Health coordinator (1)	2 (M)
	Mayor (1)	Mayor (1)	2 (M)
	Health post in-charge (1)	Health post in-charge (2)	3 (1F; 2M)
	Ward chair (1)	Public health inspector (1)	2 (1F; 1M)
Total	**11**	**11**	**22 (15F, 7M)**

F: Female, M: Male.

### Data collection

We developed separate topic guides to facilitate the interviews with FCHVs and with key informants, in English and then translated them into Nepali, and included as S1 Text. We further revised the topic guides as we collected the data and reviewed the recordings and transcripts. We collected data between December 2020 and January 2021 and focused on both lock down and post lock down periods. All the public health measures were strictly followed during the data collection. Both interviewers and the participants wore masks, maintained physical distance with frequent use of sanitizer and hand washing. We conducted the interviews in the local language in the homes of the FCHVs and the offices of the key informants. A total of 22 interviews (13 in-depth interviews with FCHVs and 9 key informant interviews) were performed. Each interview lasted between 45–60 minutes. Interviews were conducted by two researchers experienced in qualitative research with the guidance of qualitative experts and mentors.

## Reflexivity and researcher positionality

Both researchers have backgrounds in public health and social sciences and have previously worked on community-based health projects in Nepal, which provided them with a contextual understanding of the health system and gender dynamics in the study setting. Their familiarity with the local language, culture, and health programs facilitated rapport-building with participants and helped ensure culturally sensitive and contextually appropriate data collection. Researchers wrote reflective notes following each interview, took part in debriefing sessions with research team, and carried out thematic discussions and collaborative coding to make sure a range of viewpoints were taken into account.

### Data management

We recorded all interviews with participants’ consent and transcribed them into Nepali and then further translated to English by a trained translator. The research team carefully supervised the transcription and translation processes and randomly checked six translations against the Nepali transcript for accuracy.

### Data analysis

We used the thematic framework approach, informed by Morgan et al.’s gender analysis framework [37] and Steege et al.’s community health worker and gender framework [38], to analyse the qualitative data. This involves a systematic iterative process following both deductive and inductive analytical processes, where new themes emerging from the data were also included. Thematic saturation was assessed iteratively during data collection, when no new insights were identified [39], and during data analysis, when no new codes emerged from the data. The detail of the thematic framework is attached as S2 Text. The research team members first read and re-read the transcripts to familiarize themselves with the data and then developed a coding framework based on these readings as well as informed by the frameworks, study objectives and literature, which was further reviewed by senior authors. Three independent researchers coded the transcripts, progressing iteratively through continuous discussions and reflections to maintain a consistent understanding and refine the themes within the team. Initially, three coders coded the same transcripts to look for inter coder reliability and consistency. The transcripts were also re-examined where needed to ensure the reliability of coding and interpretation during the analysis. We then developed charts for each code. Finally, we used NVivo 11 pro software to support analysis.

### Ethics

We received ethical approval from the Nepal Health Research Council (Registration no. 671/2020 P) and the Research Ethics Committee of Liverpool School of Tropical Medicine (no. 20–070). We followed rigorous ethical practices, including discussing and obtaining written informed consent before conducting interviews. Participants who were unable to read and write provided a thumbprint on the consent form. We maintained confidentiality throughout the process.

### Inclusivity in global research

Additional information regarding the ethical, cultural, and scientific considerations specific to inclusivity in global research is included in the supporting information (S3 Text).

### Findings

We explored FCHVs’ experiences during COVID-19 lock down and post lockdown period and how these are influenced by gender norms and values. To set the context, in addition to their regular work, FCHVs reported performing activities specific to the COVID-19 response. In both municipalities, most FCHVs were mobilized after the lockdown was lifted. However, a few of them in Chandragiri municipality were also mobilized during the lockdown period. Their responsibility included raising awareness about COVID-19 prevention and control, such as providing information on symptoms, importance and techniques of handwashing, social distancing principles, and the use of protective equipment such as masks and sanitizers. Particularly in Gulariya municipality, which borders India, FCHVs mentioned being engaged in monitoring and reporting the arrival of external people (returnees from abroad and other districts). Their engagement varied, including working hours, and they also provided routine services to the community due to disruption in regular health services at health facilities. We present FCHVs’ experiences at different levels – family, community, and health system, with sub-themes under each level.

### Family

#### Performing dual responsibilities of home and community work.

In the context of Nepal, men are commonly viewed as primary income earners, while women are often expected to lead household chores and bear primary responsibility for unpaid domestic work. However, these norms vary across households, particularly in relation to male labour migration and geographical context, urban versus peri-urban areas. FCHVs found it challenging at times to take on work and home responsibilities and were often unable to fulfil the expectations of family members, particularly in terms of taking care of the household during pandemic. This became even more challenging when the work came up unplanned, particularly in the initial phase of pandemic, when there were no clear guidelines on the mobilization of FCHVs, and activities were just based on brief verbal instruction provided by their supervisors (health post-in-charge) over the telephone. For instance, an FCHV stated:

*They gave me a call from health post instructing me to deliver kit box to COVID patients and bringing back their health reports. Both works [farm work and duty as FCHV] coincided at the same time. There was no one at home. I asked my neighbour to prepare food for the farm worker. She [neighbour] helped me. I reached health post to receive kit box and visited COVID patient’s home.* [IDI 02, FCHV, Chandragiri]

However, most FCHVs considered it as part of their regular roles and responsibilities and thus did not consider it to be an additional burden. Most were happy to get engaged and serve the community in these challenging times and considered it as an opportunity to deliver regular responsibilities.

*While visiting community for COVID specific roles, we met other pregnant women, senior citizens suffering from [blood] pressure and sugar. The medicine for sugar and pressure is distributed for free at health post. We also asked them to visit health post to receive free medicine.* [IDI 01, FCHV, Chandragiri]

#### Balancing home and work life–support from family and self-management.

For most FCHVs interviewed, family support played a huge role in delivery of their responsibilities as community health volunteers during the pandemic. They narrated instances of support where family members helped them in completing the household chores that were usually their responsibilities, such as cooking food, and taking care of the children, as well as arranging transport for them during lockdowns when needed. A few FCHVs reported that their husbands and sons bought masks for the use of FCHVs when there was no supply from health facilities.

*If I don’t get time for household chores, I don’t need to be worried about it. My family members will take care of it.* [IDI 03, FCHV, Chandragiri]

*Senior FCHVs receive support from their husband, son, and daughter in law. At present, younger FCHVs receive help from their husbands. For example, being dropped off at health post or mother’s group meeting [to perform work responsibility]. They do not face family-related difficulties.* [KII 04, Male, Gulariya]

However, not all FCHVs received support from their families. Some FCHVs reported dissatisfaction from their husbands and elder family members, who were unhappy about the FCHVs’ involvement and inability to prepare meals due to their health-related work commitments. In one instance in Gulariya, a FCHV shared her colleague’s experience, where her husband blamed her for immoral behaviour because she carried condoms and contraceptive pills, and the demand for condoms increased during COVID-19.

*Some people feel shy; the Madhesi community cannot ask for it [condom] directly. They feel uncomfortable and initially deny the purpose of their visit. After a while, they finally tell they need condoms. I provide them in the absence of my husband to avoid suspicion. I feel uncomfortable and worry about the things [any negative things] he might say.* [IDI 07, FCHV, Gulariya]

In some cases, family members were unhappy with the FCHVs’ involvement in COVID-19 activities, particularly as these roles offered no direct benefits. Additionally, concerns were raised about the safety of both the FCHVs and their families, given the exposure to individuals with COVID-19.

Despite anxiety and fear of contracting the virus and spreading it to their family members and others, FCHVs continued their work during the pandemic. To protect their families, FCHVs mentioned taking extensive precautions, such as staying on a separate floor, washing hands, showering, and changing cloths after returning home. Interviews with FCHVs revealed that they reused masks after washing them, bought masks and other protective items with their own money due to inadequate government supply.

*How long do fifteen masks last? [Laughs] I have been buying them since then. My husband buys them in packets. How often should we buy? We get tired of buying, and cloth masks are said to be inappropriate, so we reuse them.* [IDI 01, FCHV, Chandragiri]

In addition, FCHVs have developed coping mechanisms to balance work and home life. Most FCHVs mentioned following a routine such as waking up early to complete household chores that allowed them to reach on time for their professional duties’ health facilities, community households or meetings.

### Community

#### Importance of community health volunteer’s gender in providing services.

FCHVs and key informants reported that female community health workers were valuable during COVID-19 as women have strong bonds with women and feel more comfortable in sharing their health issues, particularly those related to reproductive and maternal health. Most FCHVs perceived that having men as CTC providers could be detrimental to improving women’s health as most women may not discuss their health issues with men and men feel uncomfortable to ask about certain health issues with women.

*I feel only women can understand women’s problem. In our community, women want to talk openly with female, but if there are men [CHW], they hesitate. In my view, female is chosen [as FCHV] as there are lot of women’s health problems related to uterus and sexual diseases. Females feel comfortable to talk about the pregnancy and post-partum with female. They feel shy to communicate openly with the male.* [IDI 01, FCHV, Gulariya]

Further, most key informants and FCHVs perceived that men would not be interested in doing volunteer work, as they are socially obliged to engage in paid work.

*Men are engaged in salary-based job. They will not give time to volunteer work. Even while selecting FCHVs, employed women are not selected as FCHVs.* [KII 02, Male, Chandragiri]

Some FCHVs found it more challenging to provide services to men. Although FCHVs were native to their community and gained trust overtime, they experienced some unsupportive behaviours, particularly from men while performing their roles during COVID-19. FCHVs prioritized providing information on preventive measures to men, considering them to be at more risk due to their frequent mobility. However, FCHVs felt that men were not very receptive. They explained about a common belief that men should be in higher positions than women and that they should know more than women, which led to dismissive behaviour towards FCHVs. Men gave the impression that they already knew the information which FCHVs were providing and did not take them seriously while communicating health related messages. For some FCHVs, this behaviour was demotivating.

*Even though some people are aware, they behave badly. The males act as if they know everything... They make fun of us when we share information with them.* [IDI 06, FCHV, Chandragiri]

Most FCHVs mentioned that they were approached by women, rather than men, even in matters such as family planning that directly concerns both partners. During COVID-19, the demand and consultation for family planning services increased, partly because many men who had migrated for work returned home. However, counselling and providing family planning services to men was particularly challenging, as they often felt uneasy and reluctant to discuss these topics. In similar opinion to FCHVs a key informant mentioned,

*They (female community members) consulted FCHVs, expressing concerns about getting pregnant while already having a small child. They said, ‘the husband may or may not let [us] to use [family planning devices]. We will instead use it before they arrive.’ They used temporary contraceptive methods after consulting FCHVs.* [KII 04, Male, FCHVs’ supervisor, Gulariya]

#### Stigma related to working during COVID-19.

FCHVs were often stigmatized and discriminated against because of their engagement in COVID-19 specific work. Some women and men community members perceived FCHVs as carriers of COVID-19 due to their frequent travel and close contact with health workers and COVID-19 patients and therefore refrained from talking and sitting with them. For instance, when FCHVs made home visits, people did not respond.

*Some of them locked their door. When we rang the bell, they used to look at us and go inside fearing whether we might have brought corona. It was difficult at that time.* [IDI 05, FCHV, Chandragiri]

COVID-19 related stigma was widespread. It was not only FCHVs, but also community members with COVID-19 who were stigmatized, which hindered their healthcare seeking behaviour.

#### Community support to FCHVs during COVID -19.

Community members assisted FCHVs to identify houses where people had returned from India, abroad, or other cities. However, some people did not follow the FCHVs’ request to do a Polymerase Chain Reaction (PCR) test and stay in isolation, which made the situation more challenging. In such a situation, elected local representatives, members of the Citizen Youth’s group, and health workers supported FCHVs by revisiting those households and convincing people. Further, FCHVs mentioned receiving moral support from family members, community members, and health workers when they contracted COVID-19. One FCHV mentioned receiving regular follow-up calls with medical advice from health workers, which helped boost her self-esteem.

*In extreme difficulty, we contacted health post and the health post further coordinated with ward office. The ward office revisited households, convincing people about the need of PCR test and isolation.* [IDI 04, FCHV, Chandragiri]

### Health system support

#### Supervision and monitoring.

Most FCHVs and key informants explained that there was limited formal supervision of FCHVs work during initial and peak points of COVID-19 response and management as there was more focus on raising awareness, contact tracing, quarantine construction and regulation at the local health level. However, facility managers regularly contacted FCHVs through telephone calls or monthly meetings for updates on community situations.

*The in-charge calls us 2-3 times a week to invite for meeting at health facilities. During these calls, he used to inquire about any infected cases, or persons with cough and fever.* [IDI 01, FCHV, Gulariya]

*We usually followed up with FCHVs through phone calls to know the situation. If there was a reported suspected case at someone’s home, we conducted home visits with the help of FCHVs to verify.* [KII 01, FCHVs’ supervisor, Chandragiri]

When further exploring gendered experiences of working together, different perspectives emerged from FCHVs and key informants. Neither FCHVs nor their supervisors reported significant challenges related to gender differences, despite all FCHVs being female and most supervisors being men. However, some FCHVs expressed a preference for female supervisors, believing that this would create a more gender friendly environment and make it easier to discuss gender-sensitive topics. One FCHV shared an experience of feeling inferior when communicating with men, attributing this to deeply engrained patriarchal norms.

*We share our internal sorrows among females, but it’s challenging with Sir [male supervisors]. Expressing ourselves directly is difficult, as they are senior, and busy with several things. We wonder whether they might get angry, although our male supervisor has not shown anger.* [IDI 07, FCHV, Gulariya]

Similarly, a male supervisor expressed the view that having at least one female staff in the health facility facilitated and created a gender-friendly environment for FCHVs to talk about gender sensitive issues.

In Gulariya, a ward chairperson and FCHV supervisor were both females, and explained that it was easier for them to instruct and supervise FCHVs compared to male supervisors, as they felt more comfortable sharing personal (e.g., dysmenorrhea) and social (e.g., household responsibilities) gendered issues. In contrast, communication was more difficult with male supervisors.

*In this ward, where the chairperson is male, phone calls often go unanswered. However, the six FCHVs in this ward respond to me promptly. If I send a message to one of them, it is circulated among all. This might be because they are female, and I am also female.* [KII 02, female, Gulariya]

#### Training and supply of other Information Education Communication materials.

Most FCHVs reported that at the beginning of COVID-19, there was limited formal training and orientation on its prevention and control from municipalities, wards, and health posts. The content generally included basic information such as, COVID-19 symptoms, transmission modes, and preventive measures such as proper use of masks, gloves, sanitization, and physical distancing. As a result, most FCHVs did not receive adequate information, making it challenging for them to provide services in the community and adding stress to their work. For instance, in Gulariya, FCHVs received a short orientation by their supervisors (Health post in charge) only after lockdown ended. Whilst in Chandragiri, only selected FCHVs, working in teams formed to respond to the COVID-19 crisis, received a one-day orientation from the municipality during the initial rise of COVID-19.

However, FCHVs reported that receiving information increased their confidence and reduced their worries, knowing that COVID-19 could be prevented and cured. One FCHV from Chandragiri shared,

*Upon learning, we realized that corona is not easily transmitted. Knowing we could avoid infection and protect our community, our interest in working increased. We conducted our work without fear.* [IDI 05, FCHV, Chandragiri]

However, a few FCHVs reported not receiving any kind of training and orientation, and relied on information from social media, radios, televisions, and messages on mobile phones.

*I learned about Corona from television, news, and people in the village. I had not received any kind of training.* [IDI 05, FCHV, Gulariya]

Although creating awareness in the community was one of the key responsibilities for FCHVs during pandemic, most FCHVs explained that they did not receive adequate IEC materials for the purpose. Only some FCHVs from Chandragiri received pamphlets from the health post with pictorial and written information on hand washing and COVID-19 symptoms, which they found helpful for providing information in the community.

*The pamphlet was extremely helpful. It visually demonstrated all the symptoms of Corona, making it more useful than verbal explanations.* [IDI 01, FCHV, Chandragiri]

#### Support to FCHVs during COVID-19: supplies and remuneration.

Most FCHVs received some basic personal protective equipment (PPE) such as masks, gloves, sanitizers from health facilities and municipalities. However, the supply was uneven and inadequate for FCHVs to properly adhere to guidelines on use of these safety measures while serving communities. Thus, many FCHVs either reused the masks after washing them, bought their own supplies, or asked health facilities for more.

*I feel incredibly sad when we’re sent for work without the necessary equipment. We tell them [health workers] while visiting health post. They say it’s out of stock and promise to provide it once restocked.* [IDI 03, FCHV, Chandragiri]

The issue of inadequate supply was raised and discussed during meetings with supervisors and municipal stakeholders. However, key informants explained that due to the limited resources available in the municipalities, PPE was prioritised for health workers because of their higher exposure and proximity to infected person.

*During a shortage of PPE sets, masks, sanitizers, disinfectant spray, and infrared thermal guns, the municipality immediately brought 100 sets through special procedure. Initially, we distributed these sets to frontline health workers to boost their confidence.* [KII 04, Male, Chandragiri]

Some FCHVs, stated that inadequate supply of safety items increased risk of contracting COVID-19, with some FCHVs families not supporting, and preventing them to work in the communities during the pandemic.

One female health post in-charge (health worker) explained that PPE kits were all the same size. Therefore, the health post, in collaboration with the ward office, arranged for customized gowns for individual health workers. Additionally, different organizations such as local clubs, NGOs, INGOs, Red Cross, local organizations and individual social workers, later provided support by distributing masks, sanitizers, safety kit boxes to the FCHVs, health workers and quarantine centres.

Regarding financial incentives, most FCHVs in both Chandragiri and Gulariya reported not receiving incentives/remunerations according to the guideline “*Guidelines for Mobilization of Volunteers in the Community for the Prevention and Control of the COVID-19 Pandemic*”. A few FCHVs from Chandragiri received allowances for snacks on the days they worked, but the amount was not in accordance with the policy. Most key informants mentioned that limited local budgets hindered the provision of incentives.

*Although the community and sirs [health post in-charge and ward officials] have verbally acknowledged our work during COVID-19 response, no financial support has been provided to us for our engagement.* [IDI 02, FCHV, Chandragiri]

#### Mental health support.

Although different activities supported and helped FCHVs minimize their anxiety and fear, most FCHVs reported not receiving specific mental health support during COVID-19. However, one FCHV explained that she had regular advice and support from health workers, which increased her self-esteem.

As the COVID-19 pandemic continued, FCHVs reported that they gradually developed coping mechanisms to combat stress and anxiety. Their anxiety gradually reduced as they saw people infected with COVID-19 recover, giving the impression that COVID-19 is not always fatal. This observation provided hope and reduced their anxiety, helping them continue their work.

*At the beginning of the lockdown, we panicked, fearing infection everywhere. We feared and panicked thinking what will happen? How shall we work in the community? Overtime, we realized, we don’t need to panic, as even the COVID-infected individuals recovered.* [IDI 02, FCHV, Chandragiri]

#### Decision-making, communication process and FCHVs engagement.

Men predominantly hold higher positions or decision-making roles such as representatives of ward offices and health post in charges. A few FCHVs noted that this leads to planning and implementation processes that are not always gender inclusive and sensitive. They stated that if women are engaged in leadership positions, planning and implementation of COVID-19 activities would have been more gender sensitive, e.g., considering suitable timing for women when organizing training or other programmes.

*Yesterday, there was a programme led by ward officials. I didn’t know about it until a ward member invited me at the last hour. They should’ve informed me in the morning or afternoon so that I could have had time to manage my household chores.* [IDI 02, FCHV, Chandragiri]

Further, FCHVs mentioned that their role was often unclear and were not engaged in decision making about their mobilization during pandemic. Some FCHVs selected for the special COVID-19 response team, didn’t know why they were selected and just continued their work following the instructions from health workers and ward chairpersons.

*I was wondering why they [health post] mobilized us during COVID-19. There are other FCHVs too. Why did they mobilize only us? They usually select three of us because we are active and outspoken. But still I wondered why they selected me in such situation.* [IDI 04, FCHV, Chandragiri]

Similar to FCHVs, a female health worker in-charge of a health post shared how gender and power dynamics impacted their work. She recalled an instance, while leading contact investigation and contact tracing, she and another female colleague were not trusted and believed only male members can or should lead and handle such situations.

*One of my team members (health worker) was told by COVID-19 patient that she was too young and did not trust her because of her polite voice. Despite reassurances from the team, the patient doubted and requested another contact for conversation. Moreover, people get surprised when they know I am the boss, often expecting a male in the position.* [KII 01, Female, Chandragiri]

## Discussion

This study examines the experiences of FCHVs during COVID-19, focusing on how gender norms and values shaped their roles and how these were further influenced by the family, community, and health system levels within the Nepali context. During COVID-19, FCHVs played a vital role in awareness-raising and contact tracing. However, they struggled to balance their work with household responsibilities due to prevailing gender norms, in which men are commonly viewed as primary income earners and women as responsible for unpaid domestic and care work. These norms vary across households and have been increasingly reshaped by geographic locations, male labor migration, and changing household structures [40]. Despite these challenges, FCHVs were motivated to contribute during the crisis. While some received family support, others faced resistance, especially as their voluntary work disrupted their family and domestic responsibilities. In addition, limited PPE increased their risk of infection, leading to further family restrictions on their involvement. Moreover, limited mental health support at the health system level created additional difficulty. Although the community valued female health workers, acknowledging their importance in addressing women’s health concerns, FCHVs faced difficulties in providing services to men. This was due to societal expectations and gender roles that place men in a superior position. Our study adds to the existing literature by showing how FCHVs in Nepal adapted to expanded roles during the COVID-19 pandemic while facing persistent structural and gendered challenges.

### FCHVs role in pandemic and wider lessons for resilient health systems

In the context of pandemic control, and in keeping with other studies [25,41–43], our analysis shows that roles and responsibilities of close to community providers (CTC), particularly FCHVs in our context, expanded greatly. They became instrumental in a wide range of activities, including raising awareness, engaging in contact tracing, and supporting the community during times of illness and loss. Our study findings showed an increased demand from the community for FCHVs to provide regular routine health services like family planning during the COVID-19 pandemic. Their role became instrumental in building a resilient health system, particularly in a fragile setting where the health system faced pressures due to shortages of health workers to fulfil the pressing need and changing demand [44]. Similar roles were observed in other countries like India, where CTC providers such as traditional birth attendants and traditional healers played a significant role in addressing health issues during the pandemic [45]. Our study findings, along with those of other studies, highlight that CTC providers including FCHVs in Nepal, serve as an essential bridge between the community and the health care system, and a continued link during different emergency responses such as COVID-19, other epidemics and earthquakes [41,42]. CTC providers are often local and have built up trust with their community, which enables them to effectively communicate messages and provide services [46]. Therefore, investing in community health systems and strengthening the capacity of CTC providers through training, providing resources and incentives are essential [47].

### Gendered experiences of FCHVs, stigma and discrimination

Our study reflects that the increased responsibilities due to pandemic caused additional stress for FCHVs as they had to juggle work at home with limited support from family. This dual pressure echoes global findings [48–50] which show that women often bear a disproportionate burden due to societal expectations linked to traditional gender roles. These expectations make it difficult for female health workers to balance their domestic and professional responsibilities, while also increasing gender-specific risks and vulnerabilities such as for a higher likelihood of domestic conflict and violence [48–50]. Studies also highlighted that during COVID-19, these providers faced additional difficulties balancing work, family care, and community and health system expectations with limited support and supervision, spotlighting the impact of gender on vulnerabilities and responses [51,52]. The gendered experiences we highlight reflect the wider sociocultural context of Nepal and are not limited to the pandemic period [53]. While all findings are explored in the context of the COVID-19, their intersection with the general context demonstrates the compounded effect of shocks on already gendered roles and vulnerabilities.

Our study also highlights the difficulties FCHVs faced in providing services to men. Patriarchal norms that uphold male superiority often resulted in dismissive attitudes and resistant behaviour from men towards receiving health advice from female volunteers. This posed a critical concern during the pandemic, when rapid information needed to be disseminated to the community, including men. Similar challenges have been noticed in other countries such as Pakistan and Sierra Leone [26,54], where unsupportive attitudes from those in higher positions of power, disrespect from male colleagues, and limitations on women’s interaction with men due to social and cultural norms were prevalent [55].

Our study showed that during COVID-19, disruptions in health service delivery led to increased demand for short term family planning methods, particularly as many migrants returned home. Prevailing gender norms and discomfort posed challenges for FCHVs in providing these services to men. Similarly, findings from Kenya showed that genders norms limited men’s acceptance of services like family planning and HIV care when provided by female health workers [56]. In response to these challenges, strategies such as engaging male volunteers were implemented to address concerns about female CTC providers travelling alone to remote areas [26]. These findings highlight the need for culturally appropriate strategies that empower female CTC providers and strengthen service delivery within patriarchal contexts.

Our study illustrated how the broader gender-based constraints faced in fragile settings were further increased during COVID-19 for FCHVs who all women are as defined by the guideline. FCHVs’ engagement in COVID-19 related tasks subjected them to additional stigma and discrimination, and they were unfairly perceived as the carrier of the virus, similar to findings from studies conducted in South Asia and Africa [57]. This led to community behaviours such as avoiding interaction and discussion, and even locking doors during home visits. Such responses were particularly discouraging for FCHVs, especially when coming from men who already perceived their work as inferior with perceived low level of knowledge of FCHVs and volunteer nature of the work. These findings underscore the need to promote gender awareness not only in the roles of FCHVs but also within the broader community, by conducting community-based gender sensitization programmes, utilizing mass media and local communication channels, and integrating gender awareness into existing community programmes.

### Gender sensitive supervision and inclusivity

Our study found that FCHVs strongly preferred female supervisors, as they were perceived to be better equipped to discuss and address gender sensitive issues, helping to create a more gender-friendly environment that supports effective communication and decision-making. This preference aligns with evidence from other low-and middle-income countries, where cultural and religious beliefs influence preference for female CTC providers, allowing them to better navigate gender-related challenges more effectively and meet the needs of women [58]. Thus, strengthening the presence and role of female supervisors is needed.

Involving male volunteers alongside female in community could enhance inclusivity and shared responsibility through gender-sensitive training, community sensitization and peer networks for mixed gender volunteers [53]. However, our findings suggest that male participation in unpaid volunteer roles is less likely due to social expectations of men as breadwinners and women’s reluctance to discuss sensitive health matters with them. Male engagement as CTC providers needs careful design, that complements rather than duplicates the work of FCHVs. Encouraging male volunteers to participate in gender equity initiatives such as community awareness campaigns, joint training sessions, and advocacy for shared household responsibilities and to support their female colleagues can further promote inclusivity and a resilient health system [59].

### Implications for policy, practice and further research

Our study showed that the formal supervision of FCHVs’ work in the communities and the support mechanisms to deliver the activities were limited during COVID-19 response and management. FCHVs received minimal COVID-19 prevention training and relied on information from social media, radios, television and phones, which posed challenges to deliver community service. FCHVs had little to no incentives and financial support, inadequate mental health support and limited resources, including PPE. These constraints compounded the pre-existing challenges rooted in societal and gender norms that have shaped FCHVs’ roles and working conditions. FCHVs felt both pride in their contributions and strain from the lack of adequate compensation, highlighting how longstanding discussions over remuneration continue to shape the FCHV experience and the sustainability of community health work in Nepal [40,60]. In the post-COVID policy landscape, debates about formalizing community health volunteers roles signal potential shifts; however, the persistence of voluntary service suggests that structural challenges such as inadequate support, resources and recognition remain, and systemic change has been limited [60]. Our findings reflect the long history of Nepal’s FCHV programme, shaped by gendered expectations of unpaid care, community embeddedness, and moral responsibility. While these factors have sustained the programme, they have also normalized limited support and unpaid work, with remuneration debates remaining unresolved [61].

Gender-insensitive planning, unclear responsibilities, and gender dynamics, particularly in decision making and leadership roles, further hindered FCHVs’ work during the pandemic in our study. Addressing these challenges requires building the capacity of the community health workforce through training and awareness activities, enhancing knowledge, and establishing strong support systems to improve their competence and confidence in delivering services during crisis scenarios. In addition to technical capacity-building, it is essential to address discriminatory attitudes and behaviours within communities, while also incorporating male members, and promoting considerations of gender and equity in decision making and leadership positions. Further, during crisis, ensuring access to necessary equipment, supplies and transportation support, fair financial incentives, mental health counselling and other non-monetary rewards such as respect and work acknowledgement, can help sustain motivation, safeguard well-being and strengthen resilient community health volunteer programmes [44].

This paper highlights several policy and practice implications, as well as future research areas for strengthening the resilience capacities of FCHVs. Training and support for FCHVs should be adapted to reflect the evolving roles and responsibilities that they take on during crises. As evident in this study, community gender norms and values clearly influence FCHVs work. Therefore, policies and programmes involving FCHVs must intentionally incorporate gender and equity considerations into their design and implementation. In addition, considering the changing need and gender dimensions within the community, it is critical to understand the advantages and disadvantages of engaging male volunteers as community health volunteers, particularly in fragile settings. Developing and evaluating ways to change these norms and adapt policies to account for them is critical.

Additional research is needed to focus on how to build resilience capacities of FCHVs so that they are able to take on these roles. Further deepening and understanding the gender and inequities factors influencing the work of community volunteers, their difference in need and community perspectives towards female community health volunteers is important.

### Strengths and limitations

This paper has provided valuable insights into the lived experiences of FCHVs during the COVID-19 pandemic in Nepal. We had an embedded qualitative research team which had expertise in working with community level health staff. This is an under-researched area, and one where it is critical that evidence is generated to feed into ongoing policy and practice. However, there are several limitations. We conducted the study at one time-point during the first wave of pandemic, and our analysis and discussion is situated appropriately with the COVID-19 trajectory. Although the study is situated in one-time of COVID-19 pandemic, lessons from it may still be relevant for many other crisis situations that test the resilience of health systems. We used purposive sampling, which may have introduced selection bias, limiting the transferability of our findings. However, we ensured diversity in socio-cultural background, caste/ethnic composition, work experiences, education, and active community engagement. Although participants belonged to different ethnic groups, caste-based dynamics were not clearly discussed in the interviews. Therefore, we did not explore caste as an analytical axis. We did not include the perspectives of the community and patients, which limits triangulation from diverse perspectives, and further research is required here. Moreover, future studies should explore other intersectional lenses, such as social class, ethnicity, and caste, to provide a more complete understanding of the social and structural factors influencing the work of FCHVs.

## Conclusion

FCHVs are an integral part of Nepal’s health system and played a crucial role during the COVID-19 pandemic. Yet their experiences were shaped and constrained by gender norms and societal values, which impacted their personal, home and work life. Addressing these challenges and strengthening resilient community health systems requires gender-sensitive policies and planning, comprehensive training, equitable resource allocation, and support mechanisms at the family, community and health system levels. Additionally, promoting diversity and inclusivity in leadership positions, collaboration among stakeholders and involving male volunteers could be strategic initiatives to improve gender-inclusive community health services and enhance the resilience of FCHVs and other close to community providers programmes.

## Supporting information

S1 Table Participants detail. (DOCX)

S1 Text Interview topic guides. (DOCX)

S2 Text Thematic framework. (DOCX)

S3 Text Inclusivity in global research. (DOCX)

S1 Checklist SRQR Checklist. (DOCX)
