# Supplementary material for: The experiences of female community health workers during COVID-19 and the influence of gender norms and values in Nepal: A qualitative study
Source: PLOS Glob Public Health. 2026 May 6;6(5):e0006393. doi: 10.1371/journal.pgph.0006393 (PMC13148659; doi:10.1371/journal.pgph.0006393)
Supplement: S1 Table — (DOCX) [file pgph.0006393.s001.docx]

**Supplementary file: Participant detail**

| **Study site (n)** | **Methods (n)** | **Cadres** | **Duration of work (in years) (n)** | **Age (in years) (n)** | **Sex (n)** | **Education (n)** | **Marital status (n)** | **Ethnicity (n)** |
| --- | --- | --- | --- | --- | --- | --- | --- | --- |
| Gulariya (11) | IDIs (7) | FCHVs | ≤10 (2) | ≤30 (1) | Female (7) | Illiterate (1) | Married (7) | Brahmin (1) |
|  |  |  | 11 - 20 (4) | 31 - 40 (2) |  | Literate (1) |  | Chhetri (1) |
|  |  |  | 21 - 30 (1) | 41 - 50 (3) |  | ≤10 grade (4) |  | Madhesi (2) |
|  |  |  |  | 51 - 60 (1) |  | ≥10 grade (1) |  | Tharu (2) |
|  |  |  |  |  |  |  |  | Muslim (1) |
|  | KIIs (4) | Sub-health coordinator (COVID-focal person) | 1.5 (at Gulariya municipality) | 60 | Male | Health Assistant (HA) | Married (4) | Brahmin |
|  |  | Ward chair | 3 | 51 | Female | 10 grade |  | Brahmin |
|  |  | Mayor | 3 | 39 | Male | 10 grade |  | Madhesi |
|  |  | Health Post In-charge | 19 | 44 | Male | Auxiliary Health Worker (AHW) |  | Brahmin |
| Chandragiri (11) | IDIs (6) | FCHVs | ≤10 (2) | 31 - 40 (1) | Female (6) | ≤10 grade (5) | Married (6) | Dalit (1) |
|  |  |  | 21 - 30 (4) | 41 - 50 (4) |  | ≥10 grade (1) |  | Brahmin (1) |
|  |  |  |  | 51 - 60 (1) |  |  |  | Janajati [Newar] (3) |
|  |  |  |  |  |  |  |  | Sanyasi (Giri) (1) |
|  | KIIs (5) | Health Post In-charge | 7 | 28 | Female | Bachelors in Public Health (BPH) | Married | Sanyasi (Yogi) |
|  |  | Health Post In-charge | 6 | 27 | Male | Health Assistant (HA) | Unmarried | Madhesi |
|  |  | Health Coordinator | 22 | 53 | Male | Bachelors in Public Health (BPH) | Married | Brahmin |
|  |  | Mayor | 3 | 55 | Male | Masters in Arts | Married | Sanyasi (Giri) |
|  |  | Public Health Inspector (COVID focal person) | 25 | 55 | Male | Health Assistant (HA) | Married | Brahmin |
