# Supplementary material for: The experiences of female community health workers during COVID-19 and the influence of gender norms and values in Nepal: A qualitative study
Source: PLOS Glob Public Health. 2026 May 6;6(5):e0006393. doi: 10.1371/journal.pgph.0006393 (PMC13148659; doi:10.1371/journal.pgph.0006393)
Supplement: S1 Text — (DOCX) [file pgph.0006393.s002.docx]

# **In-depth Interview guideline**

**Topic guide**

1. **Introduction (ice breaker, keep brief)**

- Tell us a little a bit about yourself. How long have you been living here? Your education? Are you married? Do you have children?
- Can you please tell us about your role as a close-to-community (CTC) provider in general?

1. **CTC (Female community health volunteers) provider motivations**

- What were your reasons for becoming a CTC provider? (these are some examples of reasons: route into becoming formal health worker; nominated by community; role model; some were TBAs or other cadre before; financial reasons; serve their community)
- Are there more male or female workers in your cadre? Why do you think that is?

1. **FCHVs experiences of working during COVID-19**

- How did you become involved in the COVID 19 response? (were you asked, did you volunteer etc)
- What were your reasons for working as a CTC provider during COVID-19?
- Tell us about your role: what do you do / did you do? (explore in the first 3 months; after that; now; and on different phases such as complete lock down, partial lock down and others as needed)
- How often do you work and for how many hours?
- Did/ do you enjoy working as part of the COVID-19 response?
  - What did /do you enjoy the most?
  - What did / do you enjoy the least?
- What specific challenges have you faced when doing your work? What different challenges are there for women and men CTC providers? (probe about access to training, workload)
- How does your COVID 19 work affect your home, family and social life? (probe: relationships within the family, gender-based violence, decision making / negotiating about working and family care, balance between work and home life, looking after children when school closure, fear of contracting virus and passing it to family, stigma associated with being CTC provider during COVID19)
- How does your COVID 19 work affect any other responsibilities that you have (such as other work or income generating activities)?

1. **CTC providers’ experiences of management and support during the COVID-19 outbreak**

What kinds of support have you received related to your work during the COVID-19 response? (probe about the different times in the pandemic).

*Management support*

- *Training*: tell me about the training. When did you receive the training? Where did you receive the training? How long was it? Who facilitated it? What did it cover? (probe for gender considerations) What did you think of this training? Did it help you to do your work? In what ways?
- *Supervision*: tell me about the supervision you have received during COVID 19. Who has provided this? On what aspect of your work? How frequent? when was the last supervision? What did you think of this supervision? Did it help you to do your work? In what ways?
- *Equipment and supplies*: what equipment and supplies did you receive (e.g. masks, gown, hand washing stuff)? Were you able to use them and why (probe on gender)? Who provided these? When were these provided? were there any shortages? What did you do? How was this resolved? How did this affect your work?
- *Safety in doing your work:* What safety issues when working / travelling during COVID 19 did you face? How did you manage this? What support did you receive about this? How did this affect your work? are there particular safety issues that women and male CTC providers experience during COVID-19?
- *Information:* what kinds of information were you given (probe on topics e.g. use of protective equipment, handwashing)? How was this provided (e.g. handwashing posters, guidance document, messages)? How useful was this?
- *Leave for health issues including maternity:* what support did you receive for taking any leave for health issues? Did you receive pay?
- *Financial and other incentives during COVID-19:*
  - What financial incentives do you receive during COVID19? What do you think about that? (Probe: do you receive regularly, method of payment, their perceptions of the amount and whether it relates to their workload and scope; has it changed through COVID-19 lockdown/ shutdown/ quarantine)
  - Are there any differences between women and men?
  - Do you receive any other rewards and support? If so, where do they come from, on what basis do you receive them, are they fair? What recommendations do you have for incentives that would encourage you to work and continue longer as a CTC provider including during periods of shocks like C-19?
- How did the management system consider any specific needs for you as a female or male CTC providers? Probe for example, restrictions, curfews, lockdown, schools’ closure, impacts on families and how to manage/balance their home and work life during the COVID-19 response?

*Mental health support*

- What mental health issues when working / travelling during COVID 19 did you face? How did you manage this? What support did you receive about this? How did this affect your work?
- Are their particular mental health issues that female and male CTC providers experience during COVID-19? How have these been managed?

*Social support*

- What kind of support from your family have you received? How did that help you do your work?
- What kind of support from your community have you received? How did that help you do your work?

*Other support*

- Are there other people who support you in your job? Who are they and what do they do to support you? (prompt: community support, international partners support, local NGO support, other CTC providers?)
- What do you think about this support? (probe opportunities to discuss the issues and challenges faced in daily work)

*Recommendations about support*

- What recommendations do you have for managing and supporting you in your role during COVID-19?
- What recommendations do you have that would particularly support female or male CTC providers?

1. **Is there anything else you would like to add?**

**Closure and thank you.**

# **Key Informant Interview Guideline**

**Topic guide**

**1. Key informant background (ice breaker, just background information – so keep quite brief)**

- What is your current role in the health system or in the community?
- How long have you been in this role?
- Are you currently involved in the COVID-19 response? If yes, in what capacity?

**2. Range of CTC providers**

- What are the different types of close to community care providers (interviewer: provide definition of CTC provider specific to context)?
  - Are they mainly female or male? Why?
  - Probe the different types of CTC providers, when initiated and why
- Are any of them working in the COVID-19 response? If yes, in which capacity?
- What services or type of work do they provide? What are their workloads and hours of work? How have these changed throughout the time of the pandemic? How and to whom do they report?
- Which of these CTC providers do you think are the most important in the response to COVID-19?

**3. Gendered roles of the CTC providers and working at the COVID-19 frontline**

*General role of CTC providers in COVID-19*

- How did the CTC providers become involved in the COVID-19 response?
- What were the reasons for involving these CTC providers in the COVID-19 response?
- What are the challenges with involving CTC providers in the COVID-19 response? (from a management or health systems angle – e.g. skills, education level, time for this work, CTC providers’ acceptance of this type of work)

*Role of female and male CTC providers in COVID-19*

- What do the health system / facilities / managers want the (male and female) CTC providers to do during COVID-19 response? How do these roles and responsibilities differ for male and female CTC providers? (scope of care provided)
- What does the community want the (male and female) CTC providers to do during COVID-19 response? How do these roles and responsibilities differ for male and female CTC providers? (probe for context specific gendered norms; care giving roles; impact of restrictive measures on context specific gendered norms)

*Challenges faced by female and male CTC providers in COVID-19*

- What challenges do **male** CTC providers face?
  1. Probe community acceptability, travel, workloads (other work, family responsibilities etc)
- What challenges do **female** CTC providers face?
  1. Probe community acceptability, travel, workloads (other work, family responsibilities etc)

**4. Managing / supporting CTC providers during COVID-19**

*Support systems – health system*

- How has the health system (Ministry of Health, health managers, facility administration etc) responded to the challenges faced by CTC providers (captured above)?
- Have any gender related challenges in relation to the COVID-19 response been addressed? In what ways?
- How effective was this support? How has this support changed over the time of the pandemic?
- From your perspective did this address the gendered differences already discussed and in what ways?

*Support systems – Community*

- What community structures (think through examples for your context e.g. local administration, village leaders, family / household, social services) or people support the CTC providers? In what way?
- How effective is this support? Has it changed over the time of the pandemic? ?
- From your perspective did this address the gendered differences already discussed and in what ways?

*Overall experience*

- Are there any other challenges that you face in managing/supporting CTC providers? Please describe these challenges. How did you overcome them?
- What particular challenges do you face in managing/supporting female CTC providers? How did you overcome them?
- What particular challenges do you face in managing/supporting male CTC providers? How did you overcome them?
- What particular challenges does the community face in managing/supporting female CTC providers? How did they overcome them?
- What particular challenges does the community face in managing/supporting male CTC providers? How did they overcome them?

**5. Recommendations**

- Do you have any recommendations to address the challenges (that female and male CTC providers) that you have described earlier?
  - From the health system perspective
  - From the community
  - To support responses to future shocks

1. **Is there anything else you would like to add?**

**Closure and thank you.**
