# Supplementary material for: The experiences of female community health workers during COVID-19 and the influence of gender norms and values in Nepal: A qualitative study
Source: PLOS Glob Public Health. 2026 May 6;6(5):e0006393. doi: 10.1371/journal.pgph.0006393 (PMC13148659; doi:10.1371/journal.pgph.0006393)
Supplement: S2 Text — (DOCX) [file pgph.0006393.s003.docx]

**The experiences of female community health workers during COVID-19 and the influence of gender norms and values in Nepal: A Qualitative Study**

**Thematic Framework**

**Background information**

- Socio-demographic information (age, sex, gender, marital status, education, experience)

**Division of labor and roles (Who does what?)**

- **Everyday responsibilities** (general roles during COVID-19, household vs. CHW tasks, workload and burden)
- **Crisis-specific responsibilities** (COVID-specific roles, infection prevention, surveillance, contact tracing, information dissemination)
- **Selection and recruitment** (criteria, perceived reasons for recruiting women)
- **Motivations** (general and COVID-specific)

**Access to resources (Who has what?)**

- **Training and orientation** (COVID-19 training, frequency, content, usefulness)
- **Supervision** (nature, frequency, responsiveness)
- **Guidelines, IEC materials, protocols**
- **Supplies and equipment** (PPE, handwashing materials, phones, thermometers)
- **Remuneration and incentives** (allowances, bonuses, frequency, perceptions of fairness)
- **Transport/allowance, insurance, other forms of support**

**Norms and values (How are values defined?)**

- **Family and household** (support, reluctance, gendered negotiation of mobility and safety)
- **Community acceptance** (trust, recognition, respect)
- **Community stigma and discrimination** (blame, fear of infection)
- **Gendered service provision** (comfort/acceptability of female FCHVs serving men, preference for female CHWs for maternal/child health)

**Rules and decision-making (Who decides?)**

- **Household decision-making power** (permission to work, mobility restrictions)
- **Community-level decision-making** (role of local leaders, municipalities)
- **Health system decision-making** (policies, guidelines, supervision, leadership)

**Well-being and support**

- **Mental health impacts** (fear of infection, transmitting to family)
- **Psychosocial support and coping mechanisms**

**Recommendations**

- **Health system improvements** (training, remuneration, supervision, supplies)
- **Community-level improvements** (supportive norms, recognition, reducing stigma)
